# Anxious and Nonanxious Mice Show Similar Hippocampal Sensory Evoked Oscillations under Urethane Anesthesia: Difference in the Effect of Buspirone

**DOI:** 10.1155/2015/186323

**Published:** 2015-04-09

**Authors:** János Horváth, Balázs Barkóczi, Géza Müller, Viktor Szegedi

**Affiliations:** ^1^Department of Medical Chemistry, University of Szeged, Dom Square 8, Szeged 6720, Hungary; ^2^Biochemistry, Biological Research Centre, Hungarian Academy of Sciences, Temesvari Körút 32, Szeged 6726, Hungary; ^3^Biological Research Centre, Hungarian Academy of Sciences, Temesvari Körút 32, Szeged 6726, Hungary

## Abstract

Hippocampal oscillations recorded under urethane anesthesia are proposed to be modulated by anxiolytics. All classes of clinically effective anxiolytics were reported to decrease the frequency of urethane theta; however, recent findings raise concerns about the direct correlation of anxiolysis and the frequency of hippocampal theta. Here, we took advantage of our two inbred mouse strains displaying extremes of anxiety (anxious (AX) and nonanxious (nAX)) to compare the properties of hippocampal activity and to test the effect of an anxiolytic drugs. No difference was observed in the peak frequency or in the peak power between AX and nAX strains. Buspirone (Bus) applied in 2.5 mg/kg decreased anxiety of AX but did not have any effect on nAX as was tested by elevated plus maze and open field. Interestingly, Bus treatment increased hippocampal oscillatory frequency in the AX but left it unaltered in nAX mice. Saline injection did not have any effect on the oscillation. Paired-pulse facilitation was enhanced by Bus in the nAX, but not in the AX strain. Collectively, these results do not support the hypothesis that hippocampal activity under urethane may serve as a marker for potential anxiolytic drugs. Moreover, we could not confirm the decrease of frequency after anxiolytic treatment.

## 1. Introduction

Anxiety disorders are among the most abundant affective disorders, with a prevalence of 1% in the Western countries. The neurochemistry behind this set of conditions are relatively well described: the imbalance of the serotoninergic system has been implicated in regulating permanently elevated anxious mood, and a prominent class of anxiolytic drugs used in the clinic exert their action via the serotonergic system including serotonin reuptake inhibitors and agonists of the serotonin 1A receptor (5-HT1AR) [1, 2]. This receptor is most abundant in the hippocampal CA1 [3, 4], suggesting a primary role of this brain part in regulating anxiety. 

Brain oscillations are thought to govern cognitive processes, providing temporal functional linkage between different brain parts and allowing the temporal separation of functionally distinct neuronal assemblies. Among the rich repertoire of the prominent hippocampal oscillations, theta activity is implicated in various cognitive processes, including locomotion, learning and memory, and alert (fear and anxiety) [5, 6]. This rhythm is subject to serotonergic influences originating in the raphe nuclei [7–11]. Of particular interest here, almost all classes of clinically effective anxiolytics and compounds in the preclinical stage reduce the frequency of hippocampal theta activity elicited by stimulation of the reticular formation in freely behaving or anesthetized animals [12]. Notable exception includes direct bilateral histamine infusion into the lateral septum, which decreased anxiety-like responses in two models of anxiety, the elevated plus maze and novelty-induced suppression of feeding test in rats, but, instead of decreasing, the same infusion significantly increased hippocampal theta frequency elicited by reticular stimulation in urethane-anesthetized rats [13]. Similar results were reported for direct septal infusion of muscimol, which also increased theta frequency evoked by brainstem stimulation and reduced anxiety-like behaviors [14]. Moreover, if the above hypothesis holds true, then compounds that generate anxiety (anxiogenics) should increase hippocampal theta frequency. In contrast, two benzodiazepine receptor inverse agonists and an *α*2 noradrenergic receptor antagonist, all having strong anxiogenic effect, do not have any effect on hippocampal theta [15].

In this study, we took advantage of the two inbred mouse lines we breed in our laboratory to test the effect of an anxiolytic on hippocampal theta. We also tested the hypothesis that if theta frequency is in correlation with anxious phenotype, then these mice will show different theta frequency. These strains show extremes in anxiety-related behavior in the open field, elevated plus maze, and light/dark test [16].

## 2. Materials and Methods

### 2.1. Animals

Inbred mouse strains having either high or low anxiety level (AX and nAX) were bred in our animal facility. These strains were originally developed at EGIS Pharmaceuticals Co. (Budapest, Hungary) by bidirectional inbreeding based on anticipatory anxiety [16]. Male mice of 2.5–3 months were housed individually under a light/dark 12 h cycle (lights on at 08:00) at 24 ± 1°C and given ad libitum food and water. Mice were handled for 7 days prior to testing, the procedure is described by Hurst and West [13]. For the experiments, 55–59th generations were used. The study conformed to EU directive 2010/63/EU and was approved by the regional Station for Animal Health and Food Control under Project License XXXI/2012.

### 2.2. Behavior Tests

#### 2.2.1. Elevated Plus Maze

The elevated plus maze apparatus (EPM) was made of stainless steel (painted matt black) consisting of two opposite open arms (35 cm × 7 cm) and two opposite closed arms surrounded by 15 cm high walls of the same dimensions. The middle section that allows the animal to transit from arm to arm consisted of a square with dimension of 7 cm × 7 cm. The apparatus was elevated 50 cm from the floor and the open arms were equipped with 1.5 cm × 1.5 cm ledges to ensure that no animals would fall off the maze. Each mouse was placed in the central square facing an open arm and the behavior was recorded for 5 min. Before each test, the apparatus was cleared with 20% (v/v) alcohol and wiped thoroughly to eliminate the residual odor. The behavior of the mice was recorded and analyzed with an EthoVision software package (EthoVision XT 8.5 Noldus Technology, The Netherlands).

#### 2.2.2. Open Field Test

The open field (OF) arena consisted of circular shape (Ø 92 cm) plastic arena with 40 cm high black wall. Two 55 W light bulbs were positioned 50-50 cm above the center of the arena, which were the only source of illuminations in the testing room. The behavior was recorded for 5 min and analyzed offline using EthoVision. The OF arena was divided into centrum (diameter 77 cm) and periphery zones.

### 2.3. Electrophysiology

Briefly, mice were anesthetized with 1 g/kg urethane i.p. and placed in a stereotaxic frame. Under anesthesia, a multichannel electrode having 16 Pt/Ir contact points separated by 100 *μ*m (the impedance of a 15 *μ*m contact point is between 300–700 kOhm) and insulated with Formvar (Neuronelektród kft., Budapest, Hungary) was lowered into the dorsal hippocampus (AP, −2.0 mm; *L*, +2.0 mm; *H*, −2.2 mm) and a FHC concentric bipolar stimulating electrode (Bowdoin, ME, USA) was lowered in the contralateral hippocampus (AP, −2.0 mm; *L*, −2.0 mm; *H*, −5.8 mm). The location of the recording electrode was set by continuously monitoring the local field potential (LFP) activity. Similarly, the coordinates of the stimulation electrode were set to evoke the largest response. Hippocampal *θ* rhythm was evoked by a brief tail pinch. Field EPSPs were evoked by a bipolar constant current paired-pulse stimuli separated by 50 ms delivered every 20 sec with an AM Systems isolated pulse stimulator (Model 2100, Carlsborg, WA, USA). To establish baseline amplitude levels, the current used for paired-pulse stimulation was adjusted to obtain ~30% of the maximal amplitude under control conditions. One hour of recording was used to establish baseline frequency and amplitude measures followed by the injection of saline or buspirone. Theta was evoked for at least 3 times before and after i.p. injection. Evoked oscillations usually decayed after 45–90 sec., and we waited for at least 60 sec. before applying the following tail pinch. Paired-pulse stimulation was applied right before and after ≈45 min of i.p. injection.

### 2.4. Analysis and Statistics

Hippocampal LFP activity was preamplified by a Plexon headstage (Dallas, TX, USA) and amplified 100x and filtered (1–5000 Hz) with an AM Systems model 2100 differential amplifier and digitized with an A-D converter (1401 micro 3, 15-kHz sampling rate, Cambridge Electronic Design, Cambridge, UK) and commercially available software (Spike2, Cambridge Electronic Design). Power spectral analyses of hippocampal LFP were performed on 20-s sweeps following the onset of each tail pinch. Changes in power and peak frequency in response to drug treatment were assessed by paired *t*-test unless otherwise indicated. Electrophysiological features between strains were evaluated by using independent *t*-test. For behavioral data, independent *t*-test or paired sampled *t*-test was used as indicated in the Results. A *P* level of ≤0.05 was considered significant.

## 3. Results

### 3.1. Behavior

#### 3.1.1. Elevated Plus Maze

**Figure 1:**
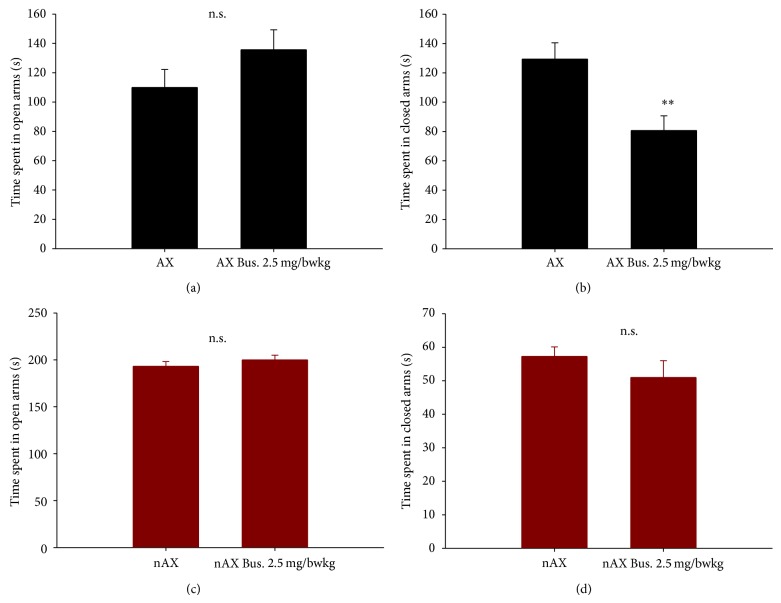
Buspirone decreased anxiety of AX mice in the elevated plus maze. A nonsignificant increase in the time spent in the open arm (a) and a significant decrease in the time spent in the closed arm (b) were induced by buspirone. There was no effect of buspirone treatment on nAX mice neither in the time spent in the open (c) nor in the closed (d) arm. ^∗∗^ *P* ≤ 0.01, *t*-test.

AX and nAX mice were subjected to elevated plus maze in order to determine their level of anxiety. There was a robust difference between AX and nAX both in the total time spent in the open (110 ± 12 sec. versus 193 ± 5;  *P* ≤ 0.001, independent *t*-test, *n* = 8, 10) and closed (129 ± 11 sec. versus 57 ± 3 sec.; *P* ≤ 0.001, independent *t*-test) arms, indicating difference in anxious phenotype. Another cohort of mice was tested 30 min following bus injection. This dose of the anxiolytic was only effective in the AX strain, as treated mice spent less time in the closed arm compared to untreated counterparts (81 ± 10 sec; *P* ≤ 0.01, independent *t*-test, *n* = 6). There was no difference between untreated and Bus treated nAX mice (200 ± 5 sec. for open arm, 51 ± 5 sec. for closed arm; *n* = 5, Figure 1).

#### 3.1.2. Open Field Test

**Figure 2:**
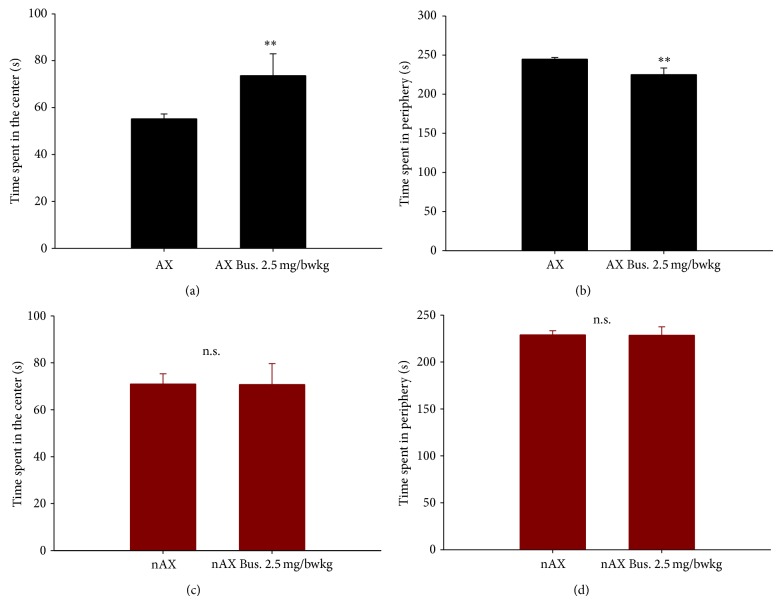
Buspirone decreased anxiety of AX mice in the open field test. An increase in the time spent in the center (a) and a decrease in the time spent in the periphery (b) were induced by buspirone. There was no effect of buspirone treatment on nAX mice neither in the time spent in the center (c) nor in the periphery (d). ^∗∗^ *P* ≤ 0.01, *t*-test.

Anxious behavior was also assessed by open field test. We observed a significant difference in the time spent in the center of the arena (55 ± 2 sec. for AX versus 71 ± 4 sec. for nAX; *P* ≤ 0.05, independent *t*-test; *n* = 33 and 28, resp.) and in the periphery (245 ± 2 sec. for AX versus 229 ± 4 sec. for nAX; *P* ≤ 0.05, independent *t*-test) between control mice. Bus treatment significantly elevated the time exploring the center (80 ± 7 sec.; *P* ≤ 0.01 versus control, independent *t*-test), while it decreased the time spent in the periphery of the arena (220 ± 7 sec.; *P* ≤ 0.01 versus control, independent *t*-test) in the AX mice (*n* = 6), a clear sign of anxiolysis. On the other hand, the same Bus treatment did not modify the behavior of nAX mice (time in center: 54 ± 6 sec; time in periphery 246 ± 6 sec; *n* = 10, Figure 2).

### 3.2. Hippocampal Theta

**Figure 3:**
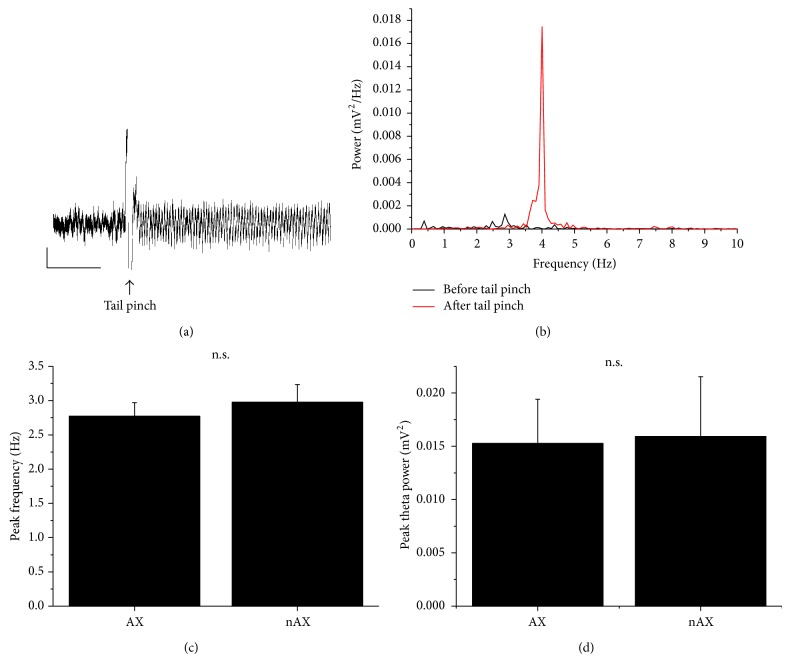
A brief tail pinch evoked a prominent oscillation (a), which was in the theta range (b). There was no difference in the peak frequency (c) and in the peak theta power (d) between AX and nAX mice. Calibration bars are 10 sec. and 0.5 mV.

**Figure 4:**
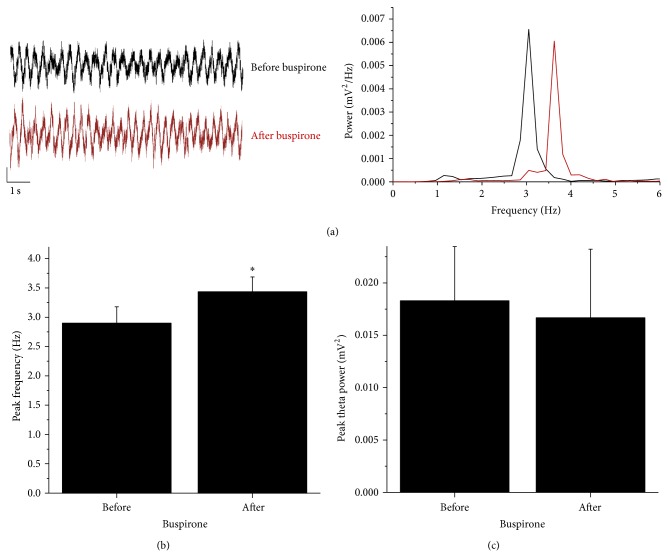
Representative local field potential traces showing activity before (black) and after (red) buspirone administration in the AX mice (a). Power spectra of the two illustrative traces indicate a peak shift after buspirone. Buspirone caused a slowing of evoked oscillation (b) but did not change the peak power of theta (c). Calibration bars are 1 sec. and 0.3 mV. ^∗^ *P* ≤ 0.05, paired *t*-test.

**Figure 5:**
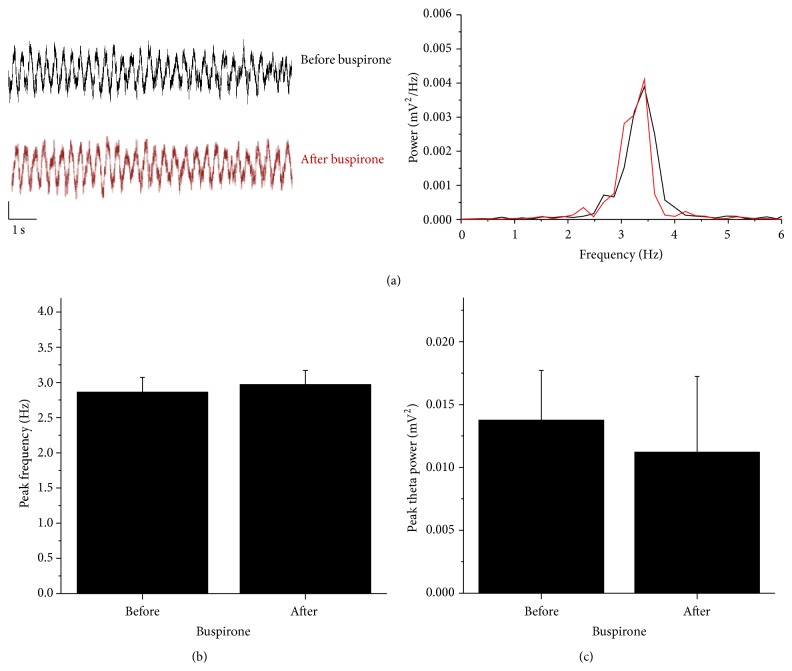
Representative local field potential traces showing activity before (black) and after (red) buspirone administration in the nAX mice (a). Power spectra of the two illustrative traces do not indicate change after buspirone. Buspirone did not change the peak frequency of evoked oscillation (b) nor did it change the peak power of theta (c). Calibration bars are 1 sec. and 0.3 mV.

Urethane anaesthetized mice were subjected to a brief tail flick which evoked prominent oscillation in the CA1 with frequency between 2.6 and 3.5 Hz. This theta activity lasted for several seconds, as is shown at Figures 3, 4, and 5. Theta activity under urethane influence has lower frequency compared to unanesthetized conditions. We did not observe any difference in the peak frequency nor in the peak power of theta between AX and nAX mice (2.7 ± 0.2 Hz for AX versus 2.9 ± 0.3 Hz for nAX; 0.015 ± 0.004 mV^2^ for AX versus 0.015 ± 0.005 mV^2^ for nAX; *n* = 11 and 11, resp.; Figure 3).

After several tail flick epochs, saline (control) or buspirone was injected intraperitoneally, and theta was evoked every 2-3 minutes. No change was seen in the oscillation following saline application (2.8 ± 0.2 Hz before and 2.7 ± 0.2 Hz after for AX; 2.9 ± 0.2 Hz before and 2.8 ± 0.3 Hz after for nAX; *n* = 5, 5, resp.; data not shown). In contrast, peak frequency continuously increased in the AX mice having received buspirone. The change peaked at 15–20 min after Bus application and remained for the rest of the recording (2.9 ± 0.3 Hz before versus 3.4 ± 0.2 Hz after; *P* ≤ 0.05, paired *t*-test; *n* = 6; Figure 4). No effect of Bus on the peak theta frequency was detected in the nAX strain (2.8 ± 0.2 Hz before versus 2.9 ± 0.2 Hz after; *n* = 6; Figure 5). Buspirone did not alter the peak power of the evoked theta neither in AX (0.018 ± 0.005 mV^2^ before versus 0.016 ± 0.006 mV^2^ after) nor in nAX (0.013 ± 0.004 mV^2^ before versus 0.011 ± 0.006 mV^2^ after).

### 3.3. Basal Synaptic Transmission and Paired-Pulse Facilitation

**Figure 6:**
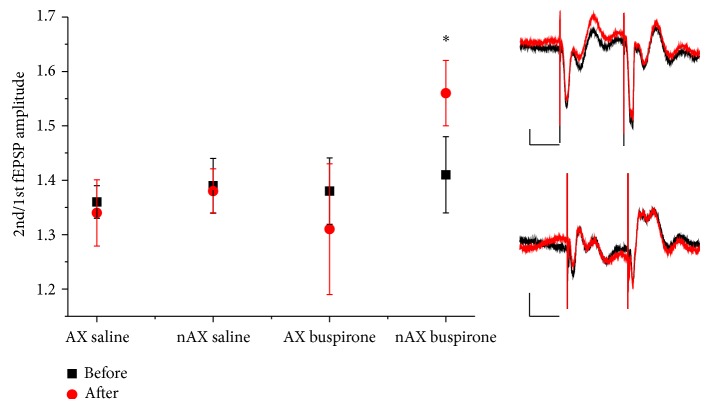
Paired-pulse facilitation remained unchanged after saline application in both AX and nAX strains. Buspirone did not alter PPF in the AX but increased that in the nAX mice. Insets show representative traces of AX (upper) and nAX (lower) paired-pulse fEPSPs recorded before (black) and after (red) buspirone. Note the slight reduction of fEPSP amplitude after buspirone. Calibration bars are 25 ms and 0.25 mV. ^∗^ *P* ≤ 0.05, paired *t*-test.

Paired-pulse recordings were taken before and after i.p. injection. Buspirone slightly decreased the amplitude of evoked fEPSPs in both AX and nAX mice, as was reported previously by O'Connor et al. [17]. We did not observe such a great reduction, as is reported in that study (around 47% for 2.5 mg/kg buspirone). Under our experimental conditions, this dose reduced fEPSPs to about 95% of the initial value. There was no difference between the two strains in this regard (94% for AX and 96% for nAX; data not shown). Similarly, no difference was found between the ratio of 2nd/1st evoked fEPSPs of AX and nAX (1.37 ± 0.04 for AX versus 1.4 ± 0.06 for nAX; *n* = 11 and 11, resp.; Figure 6). Paired-pulse facilitation (PPF) was not changed by saline injection (1.36 ± 0.03 before versus 1.34 ± 0.06 after; *n* = 5 for AX and 1.39 ± 0.05 before versus 1.38 ± 0.04 after; *n* = 5). Interestingly, we saw a decrease of PPF after Bus application in the AX strain, which was not significant (1.38 ± 0.06 before versus 1.31 ± 0.12 after; *n* = 6). On the other hand, a significant increase of PPF was detected in nAX strain following Bus administration (1.41 ± 0.07 before versus 1.56 ± 0.06 after; *n* = 6; *P* ≤ 0.05 paired *t*-test).

## 4. Discussion

Hippocampal theta activity was implicated to play a role in regulating anxious mood [6]. Enhanced theta activity can be recorded from rodents subjected to a potentially anxiety provoking situation, like the open arm of the elevated plus maze [18] and lesions of the septohippocampal pathway, which drives theta oscillation, disrupts anxiety-like behavior in tests measuring anxiety (elevated plus maze, open field, and light/dark box), social interaction, and hyponeophagia [19–21]. Serotonin can modulate theta through a number of different receptors and pathways [22–25]. Of particular interest, 5-HT1ARs are especially implicated in regulating anxiety. Mice lacking this receptor subtype show a massively elevated anxiety reaction [26–29]. Buspirone, a clinically effective anxiolytic is an agonist of 5HT1AR, and other anxiolytics, like benzodiazepines, exert their calming effects through the 5HT1AR [30]. Recently, several papers have questioned the direct correlation of hippocampal theta and anxious phenotype, suggesting that the previous correlation between anxiety level and theta frequency might be an epiphenomenon. By using our inbred mouse strains with different anxiety levels, we tested whether there is any difference in the theta oscillation parameters of these mice under urethane anesthesia.

Unlike in undrugged animals, theta oscillation emerges at a lower frequency (2.5–6 Hz) under urethane anesthesia and is sensitive to atropine or scopolamine ([31, 32] reviewed in [23]), independent of the method of evocation. This type of oscillation is referred to as type 2 theta, which, unlike the movement related Type 1 theta, is connected to sensory processing [33, 34]. Based on several reports and observation, it was proposed that nucleus pontis oralis (NPO) stimulation evoked hippocampal theta can be used as a screen for potential anxiolytic compounds, because all classes of clinically effective anxiolytics decrease theta frequency. Moreover, a hyperpolarization-activated cation (Ih) channel blocker and an antiepileptic, which decrease evoked theta but were not shown to reduce anxiety before, proved to be anxiolytic in subsequent behavioral tests [35, 36], lending additional support for the hypothesis. 

We did not observe any difference in the peak frequency nor in the peak power of theta between AX and nAX mice. Since there is a robust difference in the anxiety-related behavior between these strains, this observation does not support the straightforward connection between hippocampal theta and anxious phenotype. Moreover, the anxiolytic we have used, buspirone, affected only AX mice in the current dose and acute application (reduced anxiety) but, oddly, increased hippocampal theta frequency. We did not observe any effect on nAX mice of this low concentration of buspirone. Importantly, we have used a different kind of approach for triggering hippocampal theta, namely, tail pinch. The mechanisms behind theta evoked by either tail pinch (sensory evoked) or NPO stimulation seem to be the same. Both oscillations are modulated by the serotonergic input arising from the septum. Moreover, blockade of the caudal NPO abolishes sensory evoked theta in the hippocampus [37], suggesting that the two approaches to evoke theta share common final pathways.

Other groups have also raised concerns about the direct relationship between evoked theta and anxiety level. Compounds that enhance anxious mood (anxiogenics) failed to affect NPO evoked theta [15]. In addition, an otherwise anxiolytic treatment, histamine infusion into the brain failed to reduce NPO stimulation evoked theta, showing the lack of direct connection between the two phenomena [13]. A recent paper describes a similar phenomenon that we have found. Direct infusion of muscimol into the lateral septum increased theta frequency evoked by brainstem stimulation, but, importantly, it reduced anxiety-like behaviors [14]. It has to be noted that hippocampal theta is usually recorded from anaesthetized animals from more easily accessible dorsal hippocampus. Interestingly, electrophysiological recordings from freely moving, behaving 5HT1A KO mice, which have increased anxious phenotype, have shown that theta is not changed in the dorsal hippocampus but is elevated in the ventral part of hippocampus in a novel environment [29].

Basal synaptic transmission was slightly reduced in both strains by bus, similarly as was reported by O'Connor et al. [17] for rats. We did not see such a great fEPSP amplitude reduction as the authors did observe. A possible explanation for this contradiction might be that the mentioned study has used Wistar rats, not mice. Paired-pulse facilitation (PPF) ratio was the same in the two strains, but the effect of bus on PPF was divergent: the compound increased PPF in the nAX, while it did not modify PPF in the AX strain. The reason for this divergent effect is not clear. PPF is usually regarded as an indicator of presynaptic function; however local inhibitory networks were also shown to regulate hippocampal PPF [38]. Sibille et al. [30] have examined a decreased CA1 PPF ratio in slices taken from the 5HT1A KO animals, but this was only significant at 10 ms interstimulus interval (we have used 50 ms interstimulus interval in this study). However, the same group reported on decreased PPF in the same KO strain in the gyrus dentatus using longer interstimulus intervals (ISI, 40–90 ms), but not at 10 ms ISI [39]. Activation of the same receptor therefore might lead to increase of PPF, as was observed in the nAX animals. However, 5HT1A receptors are localized at both pre- and postsynaptic sites in the CA1; therefore, slight changes in the distribution might lead to divergent functional effects and different PPF ratio, as was seen in our mice.

## 5. Conclusion

In summary, we have found that two mouse strains having robust difference in anxiety-related behavior do not display difference in sensory evoked hippocampal theta and basal synaptic transmission. However, buspirone increased theta frequency only in AX strains, parallel to the anxiolytic effect. We found difference in the effect of buspirone on PPF between strains.

## Highlights

- Elicited hippocampal oscillation under urethane anesthesia is proposed to correlate with the level of anxiety.
- Anxious (AX) and nonanxious (nAX) mice display robust difference in anxiety level, but no difference was found in CA1 oscillation under urethane.
- Buspirone (2.5 mg/kg) is anxiolytic in the AX, but not in nAX mice.
- Buspirone increased frequency of sensory evoked oscillation in the AX, but not in the nAX.
- Paired-pulse facilitation was enhanced by buspirone in the nAX, but not in the AX strain.

## References

[1] Den Boer J. A., Bosker F. J., Slaap B. R. (2000). Serotonergic drugs in the treatment of depressive and anxiety disorders. *Human Psychopharmacology: Clinical and Experimental*.

[2] Gordon J. A., Hen R. (2004). Genetic approaches to the study of anxiety. *Annual Review of Neuroscience*.

[3] Chalmers D. T., Watson S. J. (1991). Comparative anatomical distribution of 5-HT1A receptor mRNA and 5-HT1A binding in rat brain—a combined in situ hybridisation/in vitro receptor autoradiographic study. *Brain Research*.

[4] Gross C., Zhuang X., Stark K. (2002). Serotonin_1A_ receptor acts during development to establish normal anxiety-like behaviour in the adult. *Nature*.

[5] Buzsáki G., Moser E. I. (2013). Memory, navigation and theta rhythm in the hippocampal-entorhinal system. *Nature Neuroscience*.

[6] McNaughton N., Gray J. A. (2000). Anxiolytic action on the behavioural inhibition system implies multiple types of arousal contribute to anxiety. *Journal of Affective Disorders*.

[7] Vertes R. P., Kinney G. G., Kocsis B., Fortin W. J. (1994). Pharmacological suppression of the median raphe nucleus with serotonin_1a_ agonists, 8-OH-DPAT and buspirone, produces hippocampal theta rhythm in the rat. *Neuroscience*.

[8] Kinney G. G., Kocsis B., Vertes R. P. (1995). Injections of muscimol into the median raphe nucleus produce hippocampal theta rhythm in the urethane anesthetized rat. *Psychopharmacology*.

[9] Kinney G. G., Kocsis B., Vertes R. P. (1996). Medial septal unit firing characteristics following injections of 8-OH-DPAT into the median raphe nucleus. *Brain Research*.

[10] Assaf S. Y., Miller J. J. (1978). The role of a raphe serotonin system in the control of septal unit activity and hippocampal desynchronization. *Neuroscience*.

[11] Marrosu F., Fornal C. A., Metzler C. W., Jacobs B. L. (1996). 5-HT(1A) agonists induce hippocampal theta activity in freely moving cats: role of presynaptic 5-HT(1A) receptors. *Brain Research*.

[12] McNaughton N., Kocsis B., Hajós M. (2007). Elicited hippocampal theta rhythm: a screen for anxiolytic and procognitive drugs through changes in hippocampal function?. *Behavioural Pharmacology*.

[13] Chee S.-S. A., Menard J. L., Dringenberg H. C. (2014). Behavioral anxiolysis without reduction of hippocampal theta frequency after histamine application in the lateral septum of rats. *Hippocampus*.

[14] Chee S. S., Menard J. L., Dringenberg H. C. (1831). The lateral septum as a regulator of hippocampal theta oscillations and defensive behavior in rats. *Journal of Neurophysiology*.

[15] Yeung M., Lu L., Hughes A. M., Treit D., Dickson C. T. (2013). FG7142, yohimbine, and *β*cCE produce anxiogenic-like effects in the elevated plus-maze but do not affect brainstem activated hippocampal theta. *Neuropharmacology*.

[16] Szego É. M., Janáky T., Szabó Z. (2010). A mouse model of anxiety molecularly characterized by altered protein networks in the brain proteome. *European Neuropsychopharmacology*.

[17] O'Connor J. J., Rowan M. J., Anwyl R. (1989). Serotonergic involvement in the inhibitory effects of repeated buspirone treatment on synaptic transmission in the hippocampus. *European Journal of Pharmacology*.

[18] Gordon J. A., Lacefield C. O., Kentros C. G., Hen R. (2005). State-dependent alterations in hippocampal oscillations in serotonin 1A receptor-deficient mice. *The Journal of Neuroscience*.

[19] Deacon R. M. J., Bannerman D. M., Rawlins J. N. P. (2002). Anxiolytic effects of cytotoxic hippocampal lesions in rats. *Behavioral Neuroscience*.

[20] Degroot A., Treit D. (2003). Septal gabaergic and hippocampal cholinergic systems interact in the modulation of anxiety. *Neuroscience*.

[21] Bannerman D. M., Matthews P., Deacon R. M. J., Rawlins J. N. P. (2004). Medial septal lesions mimic effects of both selective dorsal and ventral hippocampal lesions. *Behavioral Neuroscience*.

[22] Staubli U., Xu F. B. (1995). Effects of 5-HT3 receptor antagonism on hippocampal theta rhythm, memory, and LTP induction in the freely moving rat. *The Journal of Neuroscience*.

[23] Vertes R. P., Kocsis B. (1997). Brainstem-diencephalo-septohippocampal systems controlling the theta rhythm of the hippocampus. *Neuroscience*.

[24] Kitchigina V. F., Kudina T. A., Kutyreva E. V., Vinogradova O. S. (1999). Neuronal activity of the septal pacemaker of theta rhythm under the influence of stimulation and blockade of the median raphe nucleus in the awake rabbit. *Neuroscience*.

[25] Vertes R. P., Hoover W. B., Viana Di Prisco G. (2004). Theta rhythm of the hippocampus: subcortical control and functional significance. *Behavioral and Cognitive Neuroscience Reviews*.

[26] Heisler L. K., Chu H.-M., Brennan T. J. (1998). Elevated anxiety and antidepressant-like responses in serotonin 5- HT_1A_ receptor mutant mice. *Proceedings of the National Academy of Sciences of the United States of America*.

[27] Parks C. L., Robinson P. S., Sibille E., Shenk T., Toth M. (1998). Increased anxiety of mice lacking the serotonin_1A_ receptor. *Proceedings of the National Academy of Sciences of the United States of America*.

[28] Ramboz S., Oosting R., Amara D. A. (1998). Serotonin receptor 1A knockout: an animal model of anxiety-related disorder. *Proceedings of the National Academy of Sciences of the United States of America*.

[29] Adhikari A., Topiwala M. A., Gordon J. A. (2011). Single units in the medial prefrontal cortex with anxiety-related firing patterns are preferentially influenced by ventral hippocampal activity. *Neuron*.

[30] Sibille E., Pavlides C., Benke D., Toth M. (2000). Genetic inactivation of the serotonin_1A_ receptor in mice results in downregulation of major GABA_A_ receptor *α* subunits, reduction of GABA_A_ receptor binding, and benzodiazepine-resistant anxiety. *The Journal of Neuroscience*.

[31] Colom L. V., García-Hernández A., Castañeda M. T., Perez-Cordova M. G., Garrido-Sanabria E. R. (2006). Septo-hippocampal networks in chronically epileptic rats: potential antiepileptic effects of theta rhythm generation. *Journal of Neurophysiology*.

[32] Bland B. H., Colom L. V. (1993). Extrinsic and intrinsic properties underlying oscillation and synchrony in limbic cortex. *Progress in Neurobiology*.

[33] Kramis R., Vanderwolf C. H., Bland B. H. (1975). Two types of hippocampal rhythmical slow activity in both the rabbit and the rat: relations to behavior and effects of atropine, diethyl ether, urethane, and pentobarbital. *Experimental Neurology*.

[34] Vanderwolf C. H., Buzsaki G., Cain D. P., Cooley R. K., Robertson B. (1988). Neocortical and hippocampal electrical activity following decapitation in the rat. *Brain Research*.

[35] Yeung M., Treit D., Dickson C. T. (2012). A critical test of the hippocampal theta model of anxiolytic drug action. *Neuropharmacology*.

[36] Yeung M., Dickson C. T., Treit D. (2013). Intrahippocampal infusion of the Ih blocker ZD7288 slows evoked theta rhythm and produces anxiolytic-like effects in the elevated plus maze. *Hippocampus*.

[37] Kroplewski M., Orzeł-Gryglewska J., Nowacka A., Trojniar W., Jurkowlaniec E. (2010). Differential effect of procaine injection into the rostral and caudal part of the nucleus pontis oralis on hippocampal theta rhythm in urethane-anesthetized rats. *Acta Neurobiologiae Experimentalis*.

[38] Leung L. S., Peloquin P., Canning K. J. (2008). Paired-pulse depression of excitatory postsynaptic current sinks in hippocampal CA1 in vivo. *Hippocampus*.

[39] Sarnyai Z., Sibille E. L., Pavlides C., Fenster R. J., McEwen B. S., Tóth M. (2000). Impaired hippocampal-dependent learning and functional abnormalities in the hippocampus in mice lacking serotonin1A receptors. *Proceedings of the National Academy of Sciences of the United States of America*.

